# Performance Degradation Prediction Using LSTM with Optimized Parameters

**DOI:** 10.3390/s22062407

**Published:** 2022-03-21

**Authors:** Yawei Hu, Ran Wei, Yang Yang, Xuanlin Li, Zhifu Huang, Yongbin Liu, Changbo He, Huitian Lu

**Affiliations:** 1 College of Electrical Engineering and Automation, Anhui University, Hefei 230601, China; yhu@ahu.edu.cn (Y.H.); z21301130@stu.ahu.edu.cn (X.L.); hzf125521@163.com (Z.H.); changbh@ahu.edu.cn (C.H.); 2 Anhui NARI Jiyuan Electric Co., Ltd., Hefei 230601, China; weiran_nari@163.com; 3 China North Vehicle Research Institute, Beijing 100071, China; 4 JJL College of Engineering, South Dakota State University, Brookings, SD 57007, USA; huitian.lu@sdstate.edu

**Keywords:** performance degradation, degradation prediction, KJADE, LSTM, IPSO, rolling bearing

## Abstract

Predicting the degradation of mechanical components, such as rolling bearings is critical to the proper monitoring of the condition of mechanical equipment. A new method, based on a long short-term memory network (LSTM) algorithm, has been developed to improve the accuracy of degradation prediction. The model parameters are optimized via improved particle swarm optimization (IPSO). Regarding how this applies to the rolling bearings, firstly, multi-dimension feature parameters are extracted from the bearing’s vibration signals and fused into responsive features by using the kernel joint approximate diagonalization of eigen-matrices (KJADE) method. Then, the between-class and within-class scatter (SS) are calculated to develop performance degradation indicators. Since network model parameters influence the predictive accuracy of the LSTM model, an IPSO algorithm is used to obtain the optimal prediction model via the LSTM model parameters’ optimization. Finally, the LSTM model, with said optimal parameters, was used to predict the degradation trend of the bearing’s performance. The experiment’s results show that the proposed method can effectively identify the trends of degradation and performance. Moreover, the predictive accuracy of this proposed method is greater than that of the extreme learning machine (ELM) and support vector regression (SVR), which are the algorithms conventionally used in degradation modeling.

## 1. Introduction

Studies have shown that more than 45% of equipment failures in rotating machinery are caused by bearing failure [1]. The financial losses and major safety accidents that this causes in the industry can be avoided by assessing the deterioration status of equipment, which would bolster an organization’s ability to detect faults in machine bearings [2].

According to previous studies on the topic, data-driven modeling has, over time, gradually become the most effective forecasting method [3,4]. In order to predict the remaining useful life (RUL) of bearings, a large number of studies, focusing on data-driven modeling, have been carried out, including the support vector machine (SVM) and artificial neural network (ANN) [5,6]. Zheng et al. proposed the ensemble SVM for the fault detection and diagnosis of rolling bearings, in which composite multiscale fuzzy entropy was used to discern health indicators [7]. However, traditional machine learning methods like SVM require a priori knowledge of feature engineering, which is extremely difficult to implement with regard to bearings due to the complex working conditions they operate under. Deep learning-based algorithms provide an alternative solution to this problem [8,9,10]. Chen et al. proposed a method based on neuro-fuzzy systems (NFSs) and Bayesian algorithms, which use trained NFSs as predictors to discern the degradation of a given machine’s fault state over time [11]. Ren et al. proposed the use of the spectral principal energy vector method in obtaining bearing signal feature vectors. They adopt the deep convolutional neural network to predict the RUL of rolling bearings [12]. The recurrent neural network (RNN), as an important subfield of deep learning, performs well with regarding time series processing because the RNN can forecast using all available historical data [13]. Malhi et al. made further strides towards putting forth a long-term prediction model for machine condition monitoring based on RNN [14]. However, the disappearance, or even the explosion, of gradients during network training seems to restrict this method’s applicability [15].

To solve the issue, Hochreiter and Schmidhuber presented a variant of the RNN network in 1997, namely, the long short-term memory (LSTM) neural network, which addressed the problem by adding a gating mechanism [16]. At present, LSTMs are widely used in a variety of different fields, such as speech recognition, time series modeling, video classification, traffic flow prediction, and so on. Besides this, the LSTM method has also been used to predict bearing degradation, exploring the correlation between bearing degradation data and time [17]. Liu et al. proposed the use of an end-to-end model in predicting the degradation trends of bearings. His model used CNN for data reduction and feature extraction and a LSTM for time series processing [18]. Elsheikh et al. proposed bidirectional rocking long-term and short-term memory to predict the RUL of turbofan engines [19]. Tang et al. used a stacked automatic encoder (SAE) to obtain the bottleneck characteristics of bearing signals and predicted bearing performance degradation with a LSTM [20].

In application, the choice of the network structure, the number of hidden layers, and the learning rate setting will significantly influence the predictive capability of LSTMs [21]. Typically, the complex structure and parameters of LSTM neurons are mostly determined by experience, or by multiple parameter adjustments with expensive time, which involves a lot of randomness and reduces the model’s predictive capability. Therefore, a set of hyper-parametric optimization algorithms were developed to select the parameters automatically. The particle swarm optimization (PSO) algorithm is commonly used for model parameter optimization in the field of bearing performance degradation assessment [8,22,23]. However, the traditional PSO algorithm suffers from slow convergence as well as local optimization problems, which affects the performance of the model. Hence, a modified PSO algorithm is suggested for the purpose of optimizing the LSTM model’s parameters. The modified IPSO-LSTM module was applied to predict bearing performance degradation trends.

## 2. Methodology

### 2.1. LSTM

The mechanical degradation process, for example, on a rolling bearing, is a process of accumulation and continuous fault development [24]. Its degradation is determined by assessing its currently observable state as compared with its state in the recent past. The traditional neural network only uses the most recently documented state for its model, making it difficult to characterize deterioration and performance degradation over time. The LSTM is a type of RNN. An RNN is a neural network that handles sequential data and can be used to connect information from the recent past to the current task. However, as the distance between relevant information and the information taken from the past increases, the RNN loses its ability to learn and use distant details. Multiple control gates have been designed to replace the RNN in order to solve this problem. Thus, the LSTM network is constructed [16].

**Figure 1:**
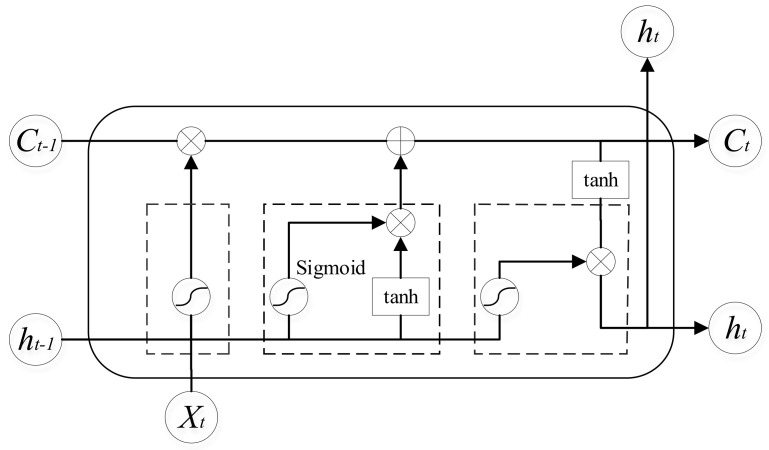
Structure of long short-term memory hidden unit.

The LSTM solves the problem of gradient disappearance and explosion through the use of the aforesaid gates. In the LSTM structure, $f_{t}$, $i_{t}$, and $o_{t}$ are three gates, which are designed to control the flow of information. $f_{t}$ controls the information of memory cells from time *t*-1 to time *t*. $\;i_{t}$ controls the information input to the memory cells at time *t*, and $\;o_{t}$ controls the information of memory cells at time *t* to the hidden state of $h_{t}$.

(1) $$f_{t}=\sigma (w_{fc}C_{t-1}+w_{fh}h_{t-1}+w_{fx}x_{t}+b_{f})$$

(2) $$i_{t}=\sigma (w_{ic}C_{t-1}+w_{ih}h_{t-1}+w_{ix}x_{t}+b_{i})$$

(3) $$o_{t}=\sigma (w_{oc}C_{t-1}+w_{oh}h_{t-1}+w_{ox}x_{t}+b_{o})$$

where $w_{fc}$, $w_{ih}$, and $w_{oh}$ are the weight matrix between gate $f_{t}$ and memory cell $C_{t-1}$. $b_{f}$ is the bias of the gate $f_{t}$. Other weight matrices are derived from the following: $C_{t}$ and $C_{t-1}$ represent the values of memory cells at time *t* and time *t*-1. $b_{f},\;b_{i}$, and $b_{o}$ represent the bias. σ is the activation function. The hiding unit structure of the long and short-term memory network is shown in Figure 1.

The LSTM can predict degradation due to the time-varying characteristics of performance degradation and the advantages of LSTMs in modeling and forecasting time series. However, the structure of the LSTM model is complex. Some parameters need to be set synthetically, such as the time frame, the batch size, the number of hidden layer units, etc., which makes it difficult to meet the highly precise requirements for predicting time series degradation. Thus, it is necessary to find the optimal model parameters for each iteration in order to maintain strong predictive accuracy. This optimal model is realized through the use of a swarm intelligence algorithm, which auto-selects and optimizes the LSTM model’s parameters to improve the prediction.

### 2.2. IPSO

A particle swarm optimization (PSO) algorithm is a population intelligent optimization algorithm used to simulate birds’ foraging behavior. Kennedy and Eberhart first proposed it in 1995 [25]. A standard particle swarm optimization algorithm sets the particle swarm size as *m*, and each particle has an *n* dimension search region. $x_{i}=\left(x_{i1},x_{i2},x_{i3},\ldots ,x_{in}\right)$ represents the search position of particle *I* in space.$\;v_{i}=\left(v_{i1},v_{i2},v_{i3},\ldots ,v_{in}\right)$ is the velocity of the particle, *i,* which represents the moving distance of the particle in each position update. $p_{i}=\left(p_{i1},p_{i2},p_{i3},\ldots ,p_{in}\right)$ records the search optimal bit value of the particle, *i*. $p_{g}=\left(p_{g1},p_{g2},p_{g3},\ldots ,p_{gn}\right)$ is the optimal particle location in the current population. In a traditional PSO algorithm, the positions and velocities of particles are updated through Equations (4) and (5).

(4) $$v_{ij}(t+1)=wv_{ij}(t)+c_{1}R_{1}(p_{ij}(t)-x_{ij}(t))+c_{2}R_{2}(p_{gj}(t)-x_{ij}(t))$$

(5) $$x_{ij}(t+1)=x_{ij}(t)+v_{ij}(t+1)$$

where w is the inertia weight factor, and the range of w is (0, 1.4); $c_{1}$ and $c_{2}\;$ are learning factors; $R_{1}$ and $R_{2}$ are random numbers between 0 and 1; $v_{ij}\left(t+1\right)$ is the *j* dimension velocity component of the particle, *i,* in the *t*+1 iteration; $x_{ij}\left(t+1\right)$ is the *j* dimension position component of the particle, *i,* in the *t*+1 iteration; $p_{ij}\left(t\right)$ is the *j* dimension optimal position component of the particle, *i*, in the *t* iteration; $p_{gj}\left(t\right)$ is the *j* dimension position component of the optimal solution in the population in the *t* iteration; 1≤i,g≤m, 1≤j≤n.

However, the disadvantages of a PSO algorithm include low convergence accuracy and premature results. The diversity of the population decreases while the iteration times increase, the algorithm falls into the local optimal solution and the algorithm prematurely solves an incomplete problem. To solve this problem, the position updating model and parameter adjustment strategy of the particle swarm are modified.

1. Parameter adjustment strategy

The inertia weight, w, has a significant influence on the performance of particle swarm optimization. In the early stages, a strong search ability is needed to search for the best information quickly. And in the late stages, a fine selection is required to search for accuracy.

(6) $$w^{t}=w_{\max}-t*(w_{\max}-w_{\min})/t_{\max}$$

where $w_{\max}$ and $w_{\min}$ are the upper and lower limits of the preset inertia weight, and in general, $w_{\max}=0.9$ and $w_{\min}=0.4$, respectively; $t_{\max}$ is the maximum number of iterations.

In the traditional PSO algorithm, $c_{1}$ and $c_{2}$ are fixed values. The improved $c_{1}$ and $c_{2}$ can adaptively adjust learning factors and inertia weight. To find the optimal solution, independent and team learning abilities are adjusted in different search times.

(7) $$c_{1}=c_{\max}+(c_{\max}-c_{\min})(1-{\left(e^{-w}-1\right)}^{2})$$

(8) $$c_{2}=c_{\max}-(c_{\max}-c_{\min})(1-{\left(e^{-w}-1\right)}^{2})$$

2. Particle swarm position updating model [26]

In the early stages of the searching process, particles have a strong self-learning ability, and the search iterative step size should be set to large. With the search time increasing, the space range of solutions becomes smaller. To search for accurate solutions, the search iteration step size of particles should be reduced accordingly. Therefore, an adaptive adjustment factor, μ, is added to the particle position updating algorithm.

(9) $$\mu =1/(1+e^{-t/t_{\max}})+1/2$$

where *t* is the iterations.

The improved particle position update formula is then as follows.

### 2.3. IPSO-LSTM

As can be seen in Section 2.1, due to the advantage of processing time sequences, a two-layer LSTM is used as the backbone network for the high dimensional degradation feature extraction in this paper. The hidden state of each time step in the first layer is retained to serve as the input of the second layer, which only returns the hidden state of the last time step. To avoid model overfitting, a dropout regularization strategy is employed after each LSTM layer. Then, the learned representation features are fed into the fully connected layer to be mapped into a one-dimensional degeneration metric.

First, the hyper-parameters that need to be determined for the LSTM’s backbone network include the number of hidden nodes in the first second layer. The hidden layers play a vital role in extracting high-dimensional features and internal laws. The model’s performance is affected mainly by the number of hidden nodes. Too many nodes will increase the training time and may lead to overfitting. Too few will reduce the model’s learning ability to the extent that the sparse adequate information extracted will not suffice in solving the problem. Therefore, the model structure’s complexity and predictive accuracy should be considered comprehensively in selecting the number of nodes when designing the network.

In addition, most neural networks are usually optimized by a gradient descent algorithm. The gradient descent is calculated as follows:

(10) $$g=\frac{1}{m}\nabla_{\tilde{\theta }}\sum_{i}L(f(x^{\left(i\right)};\tilde{\theta }),y^{\left(i\right)})$$

where *m* is the batch size; $y^{\left(i\right)}$ is the target corresponding to *m*; θ is the updated parameter; f is the random target function with the parameter, θ.

As seen from Equation (10), increasing the batch size reduces the gradient and makes the gradient more accurate. This indicates that *t* stability of the convergence is enhanced by increasing batch size in the correct range.

As described above, it is clear that the three hyper-parameters, namely the number of hidden nodes in the first LSTM layer, the number of hidden nodes in the second LSTM layer, and batch size, are the key factors affecting the performance of the model. The specific representations and ranges are shown in Table 1. In this paper, the IPSO algorithm is used to optimize and automatically select the parameters of the LSTM model.

**Figure 2:**
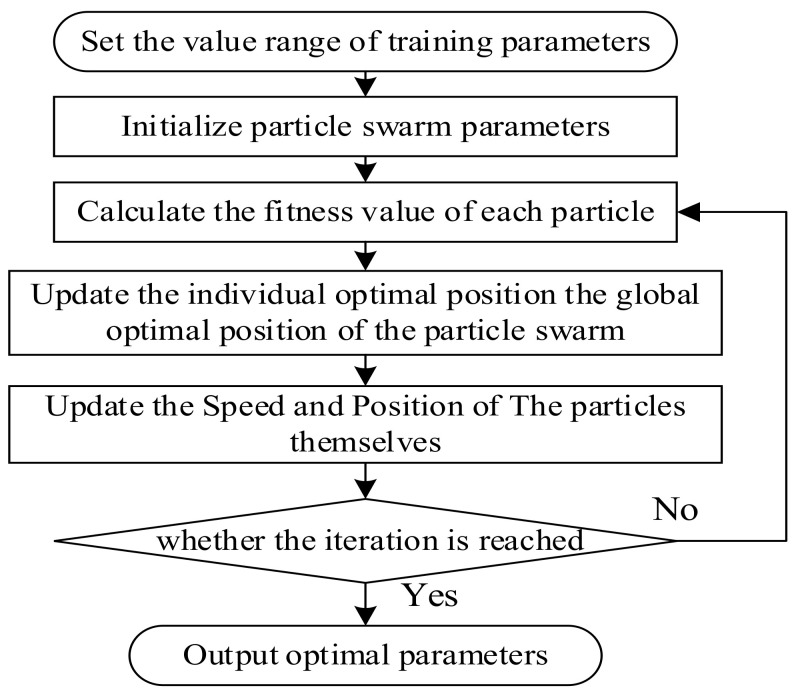
Parameter optimization flowchart.

$h_{1}$$h_{2}$ The flowchart of LSTM parameters optimized by IPSO is shown in Figure 2. The steps are as follows:

1. Initialize the parameters. Determine the population size range, iteration times, learning factors, location, and velocity;
2. Initialize the position and velocity of the particles. Generate the population particles $X_{i,0}\left(h_{1},h_{2},Sm\right)$ randomly. Where $h_{1}$ and $h_{2}$ denote the number of neurons in the first and second hidden layer, respectively, and Sm represents the batch size;
3. Determine the evaluative function of the particles. The particle $\;X_{i,0}$, in step 2 above, is assigned to the LSTM parameter. The data are partitioned into the training samples, validation samples, and test samples. The fitness value, fit, of individual $X_{i}$ is defined as the target function, which is set as:
  (11) $$fit=\sqrt{\frac{1}{n}\sum_{i=1}^{n}{\left({\hat{y}}_{i}-y_{i}\right)}^{2}}$$
  where ${\hat{y}}_{i}$ is the predicted value; $y_{i}$ is the actual observation;
4. Calculate the fitness value of each particle position, $X_{i}$. Individual extreme value and the population extreme value are determined according to the initial particle’s fitness value, and each particle’s best position is taken as its historical best position;
5. Update the velocity and position of the particle;
6. Determine whether the end condition of the iteration has been met. If it has, output the optimal parameter; Otherwise, go to step 4 to continue the iteration.

## 3. Results IPSO-LSTM Method for Bearing Performance Degradation Prediction

In most practical industrial applications, the actual working conditions of mechanical equipment are complex and dynamic. Bearing vibration signals collected by sensors contain rich information. A single feature cannot fully describe the state of bearing vibration signals. The kernel joint approximate diagonalization of eigen-matrices (KJADE) is to map the observation data $X=\left\{x_{1},x_{2},\ldots ,x_{m}\right\}$ to a high-dimensional feature space *F* through a nonlinear function Φ, and the mapped feature space is $F=\left\{\Phi \left(x_{1}\right),\Phi \left(x_{2}\right),\ldots ,\Phi \left(x_{M}\right)\right\}.$ The inner product of two vectors in the feature space is calculated following the kernel function, and an m×m kernel matrix *K* is established as follows:

(12) $$K_{ij}=k\left(x_{i},x_{j}\right)=\langle \Phi (x_{i})\cdot \Phi (x_{j})\rangle$$

where $x_{i}$ and $x_{j}$ are the sample vectors. Therefore, the KJADE algorithm is employed to characterize the bearing degradation state.

**Figure 3:**
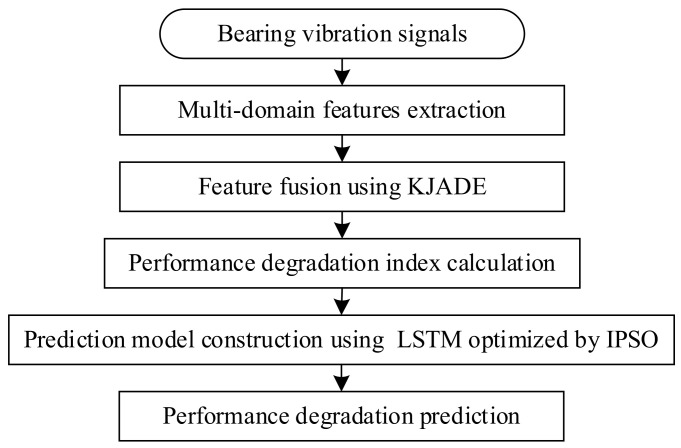
Performance degradation prediction by IPSO.

The step flow chart of the method is shown in Figure 3. The operations are described as follows:

1. Original feature extraction. The full life vibration signal of bearing is analyzed in both the time and frequency domains to avoid the insufficiency of single feature evaluation ability. Eight features in time-domain and frequency-domain are extracted to form a high-dimensional feature vector, as shown in Table 2. *T*1–*T*8 are the mean value, root mean square (RMS), absolute average, skewness, waveform index, impulsion index, and kurtosis index, respectively. Among others are frequency domain features, where $s_{i}$ is a spectrum for i=1,2,…,N (*N* is the number of spectrum lines) and $f_{i}$ is the frequency value of the *i*-th spectrum line, indicating the degree of dispersion or concentration of the spectrum and the change of the dominant frequency band;
2. KJADE features fusion. Considering the nonlinear characteristics of bearing vibration, the redundancy of the original multi-domain degradation characteristics, and some features that are not sensitive to the bearing degradation state, it is necessary to fuse multi-domain features. Therefore, the KJADE algorithm based on kernel function is employed to extract more effective, but low-dimensional, degradation characteristic indexes [27]. KJADE has better nonlinear processing capabilities for bearing vibration signals. It maps the observation data to a high-dimensional feature space through a nonlinear function. Then the JADE can be used in this feature space to change the nonlinear separable problem into a linear one;
3. Degradation assessment index calculation. The vibration signal collected at the beginning of the bearing operation is taken as the health state, corresponding to the health sample after dimension reduction. The subsequent signal is selected as the monitoring sample. To quantify the dispersion between monitoring and health samples and the aggregation between different classes, the evaluation factor, SS, composed of between- and within-class scatter matrix, is used as the performance degradation index [12];
4. IPSO-LSTM model construction. The number of hidden layer nodes and the batch size of the LSTM neural network are taken as optimization objects. The LSTM is constructed according to the corresponding parameters of each particle. The IPSO algorithm is used to acquire the optimal hyper-parameter set for each iteration automatically;
5. Predicting the performance degradation. The LSTM model is constructed with the optimal value of hyper-parameters, and the bearing data is used as input for training and prediction.

## 4. Case Analysis

### 4.1. Case 1

**Figure 4:**
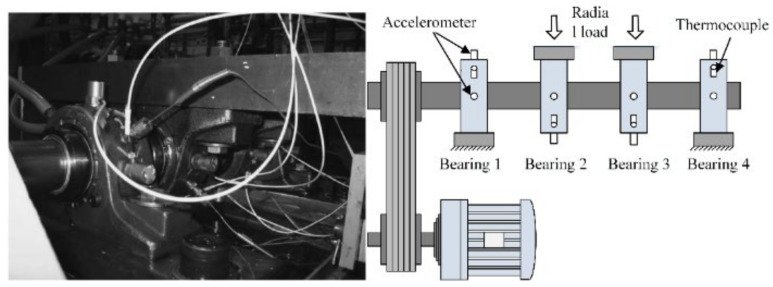
Experimental setup.

The Intelligent Maintenance System (IMS) Center of the University of Cincinnati’s full-life vibration signals of bearings are used to confirm the proposed method [28]. The experimental platform is shown in Figure 4.

**Figure 5:**
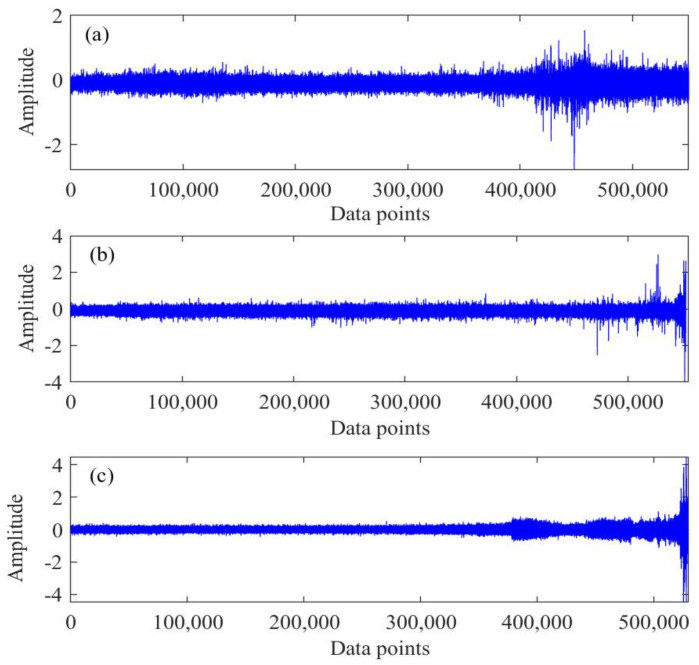
Exemplary diagram of bearing vibration data: (**a**) rolling fault; (**b**) inner fault; (**c**) outer fault.

The bearing type is ZA-2115, and the experimental conditions were as follows: output speed was 2000 rpm, the radial load was 6000 lbs, and the sampling frequency was 20,480 Hz. A total of 984 sets of vibration signal data were recorded. The whole experiment was completed in three groups. By the end of the experiment, an inner fault in bearing 3 and a rolling fault in bearing 4 were observed in the first group. An outer fault in bearing 1 in the second group and an outer fault in bearing 3 in the third group were also observed. Among them, the rolling fault and inner fault in the first group, along with the outer fault in the second group, were selected as objects for analysis. The corresponding vibration data of life is shown in Figure 5.

Based on the method in Section 2, the IPSO algorithm is used to optimize the LSTM model’s predictive parameters. The initial parameters of the IPSO are as follows: the number of particles is 10, the dimension of particle swarm is 3, the maximum velocity of the particle is 1, and the maximum iteration number is 50. The range of particle locations, namely the number of hidden layer nodes, is set to (100, 300), and the batch size is (30, 200). The upper and lower limits of the inertia weight are *w*_max = 0.9 and *w*_min = 0.5, while the upper and lower limits of the initial learning factors *c*_max and *c*_min are 2 and 1, respectively. These are the optimal parameters obtained by comparative experiments. In this study, the first 60% of the performance data is used as the training set, and 20% of the rest is saved as a validation set. Besides this, the model is optimized by an Adam algorithm, and the root mean square error (RMSE) is applied as the target criteria.

**Figure 6:**
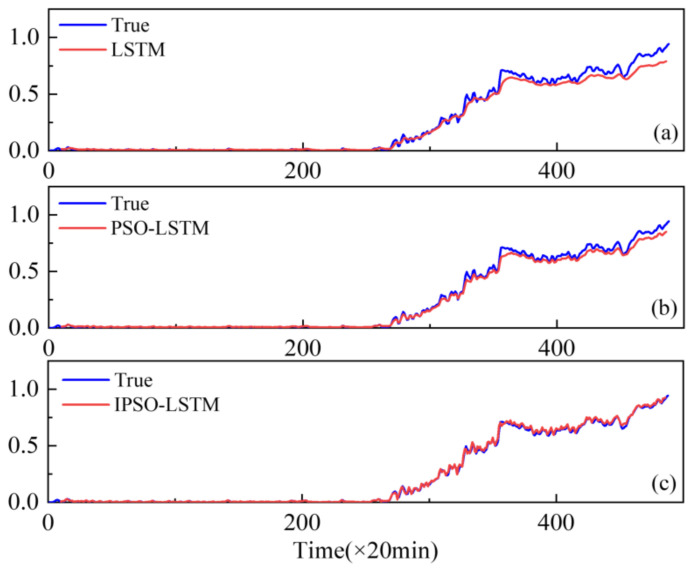
Performance degradation predictions for outer bearings for the: (**a**) LSTM; (**b**) PSO-LSTM; (**c**) IPSO-LSTM.

**Figure 7:**
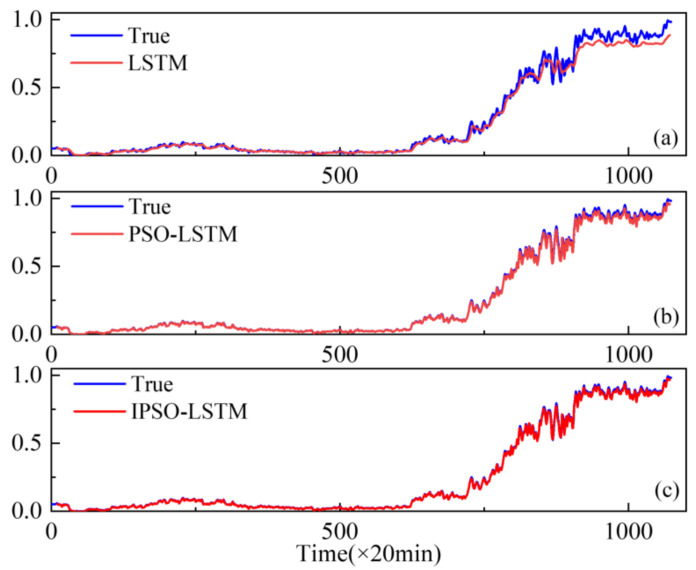
Performance degradation predictions of roller bearings for the: (**a**) LSTM; (**b**) PSO-LSTM; (**c**) IPSO-LSTM.

**Figure 8:**
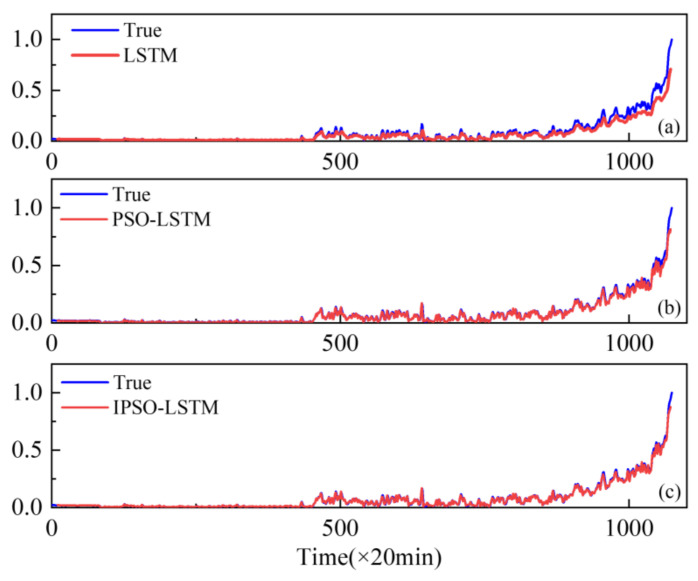
Performance degradation predictions of inner bearings for the: (**a**) LSTM; (**b**) PSO-LSTM; (**c**) IPSO-LSTM.

To demonstrate the superiority of the proposed method, the performance of conventional LSTMs and PSO-LSTMs have been compared. The resulting real degradation trends, which can be expressed as a degradation index, are obtained via feature fusion using the KJADE algorithm. Additionally, the comparison results of the degradation trends predicted by each model are shown in Figure 6, Figure 7 and Figure 8, where the y-axis is the degradation index. In addition, RMSE is used as an additional metric to measure the performance of the model, with the results shown in Table 3. The RMSE calculation is shown in Equation (13).

(13) $$\mathrm{RMSE}=\sqrt{\frac{1}{n}\sum_{i=1}^{n}{\left({\hat{y}}_{i}-y_{i}\right)}^{2}}$$

where ${\hat{y}}_{i}$ is the predicted value; $y_{i}$ is the actual observation; *n* is the total number of samples in the faulty bearing.

From Figure 6, Figure 7 and Figure 8, it can be seen that our proposed IPSO-LSTM method tracks the degenerate states significantly better than the other two methods in all three failure modes, especially the LSTM method without the hyper-parameter optimization process. In terms of quantitative metrics, the RMSE results in Table 3 also illustrate the superiority of the proposed method.

**Figure 9:**
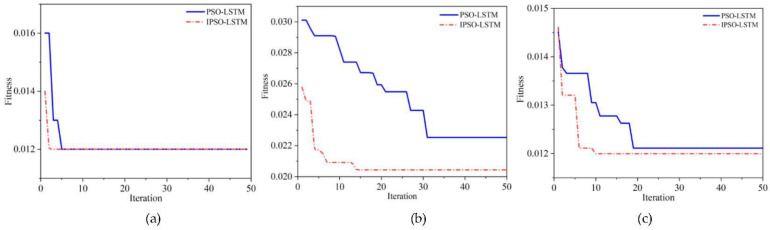
Optimization iteration results for the: (**a**) outer bearing; (**b**) roller bearing; (**c**) inner bearing.

The above results show that the IPSO algorithm is effective in optimizing the hyper-parameters of the LSTM based network, which can automatically and accurately search for the optimal parameters. To further illustrate the advantages of the IPSO algorithm in optimizing speed and avoiding local extremum, we visualize the parameter search processes, which are shown in Figure 9.

Overall, the convergence speed and fitness of the IPSO algorithm are better than the traditional PSO algorithm. Specifically, as Figure 9b,c demonstrate, IPSO has good optimization ability and can quickly find the optimal global point. Compared with the PSO, the IPSO algorithm has a faster convergence speed. Figure 9a shows that although the final fitness error is the same, the IPSO algorithm converge is faster.

**Figure 10:**
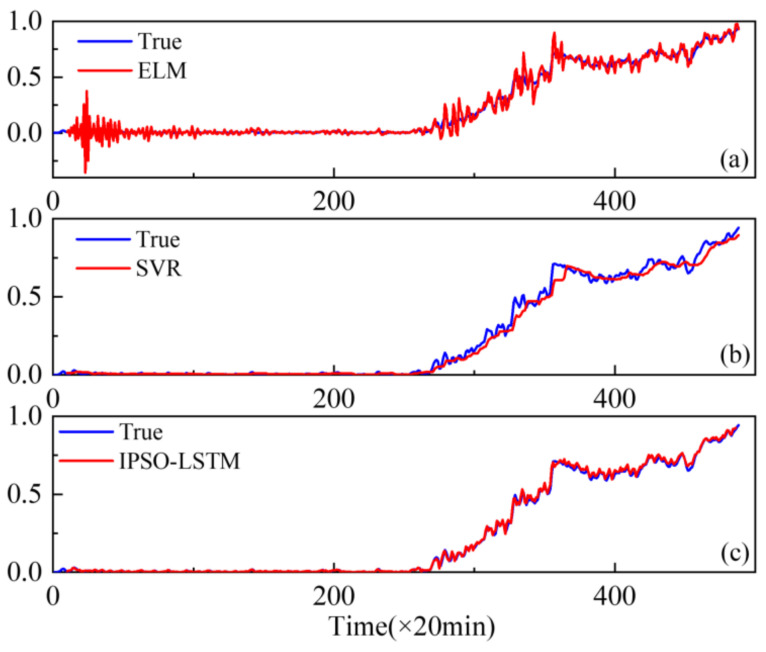
Performance degradation predictions of the outer bearings for the: (**a**) ELM; (**b**) SVR; (**c**) IPSO-LSTM.

**Figure 11:**
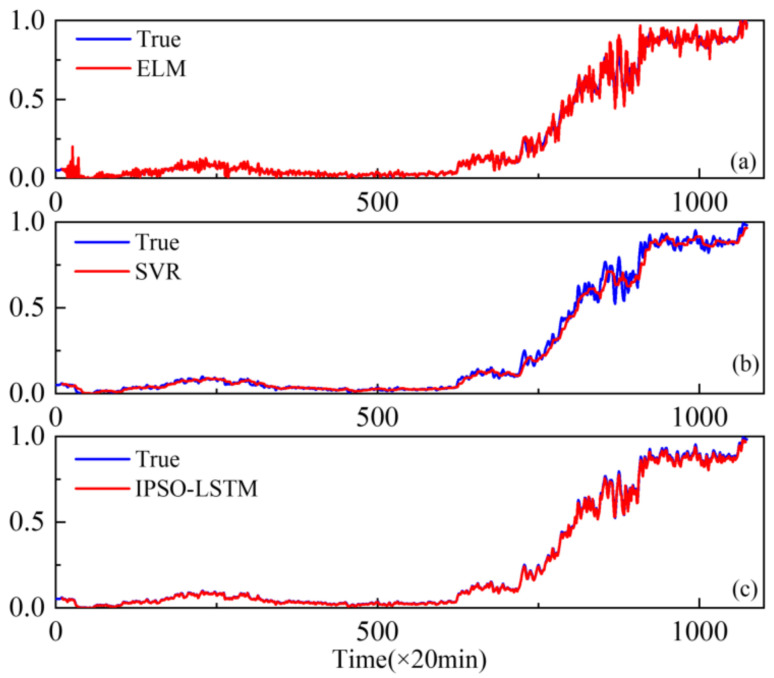
Performance degradation predictions of roller bearings for the: (**a**) ELM; (**b**) SVR; (**c**) IPSO-LSTM.

**Figure 12:**
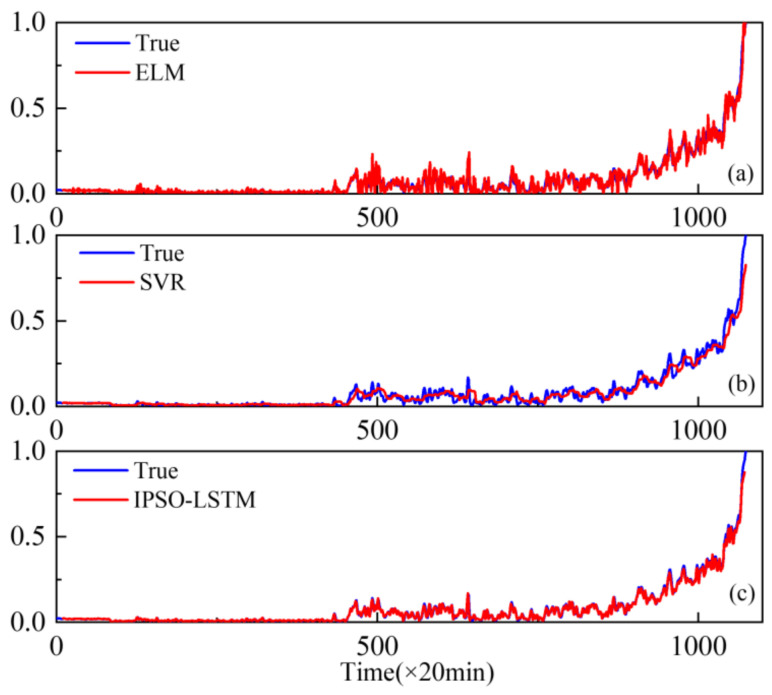
Performance degradation predictions of the inner bearings for the: (**a**) ELM; (**b**) SVR; (**c**) IPSO-LSTM.

Furthermore, extreme learning machines (ELM) and support vector regression (SVR), which have been widely used with good performance degradation prediction [29,30], are compared with the proposed IPSO-LSTM for effectiveness. The comparison results are shown in Figure 10, Figure 11 and Figure 12.

The results show that the prediction results of the IPSO-LSTM method are more in line with the original curve, with greater predictive accuracy. This is demonstrated in the RMSE values in Table 4. Predictive errors in the proposed method are minimal, which verifies the effectiveness of the proposed IPSO-LSTM method.

### 4.2. Case 2

**Figure 13:**
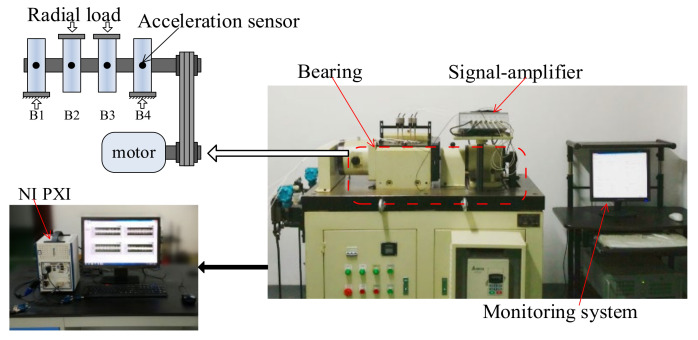
Experimental setup.

The lab experiments used four HRB6305 bearings. They were fixed on the same shaft and connected with the motor. A radial load of 750 kg was applied to all bearings to accelerate the bearing damage process, and the bearing speed was 3000 rpm. Full-life vibration signals were obtained by the NI PXI acquisition system. The vibration signals acquisition frequency was 20 kHz, the data were collected every 5 min. The experimental platform is shown in Figure 13.

**Figure 14:**
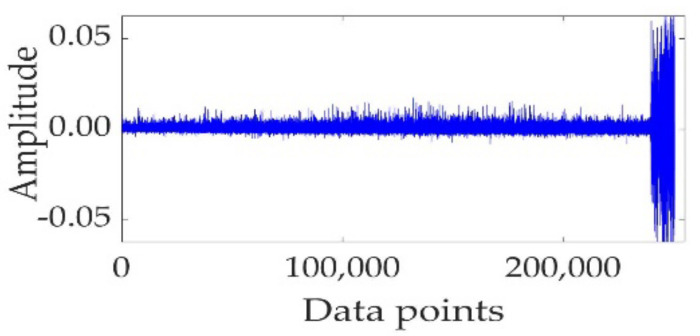
The full-life original vibration signal.

**Figure 15:**
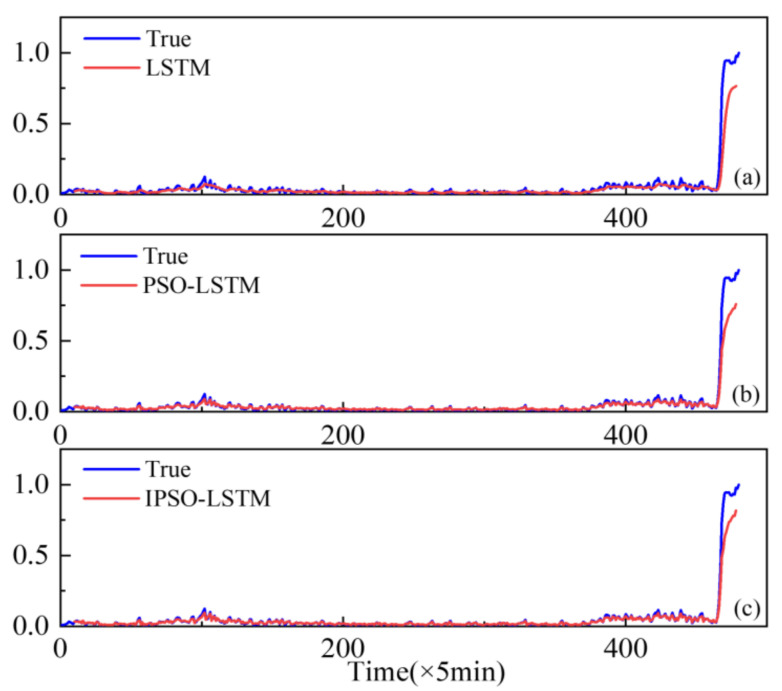
Performance degradation predictions of the roller bearings for the: (**a**) LSTM; (**b**) PSO-LSTM; (**c**) IPSO-LSTM.

The fault in the rolling element is taken as the experimental object. Figure 14 shows the full-life original vibration signal of the rolling element. The mixed-domain features are extracted from the bearing data. KJADE is used for feature fusion to acquire an optimal feature parameter set, and the SS is calculated from fusion features to obtain the degradation index. The proposed method is used to predict the performance degradation and compared with the LSTM and PSO-LSTM methods. The prediction curve is shown in Figure 15.

**Figure 16:**
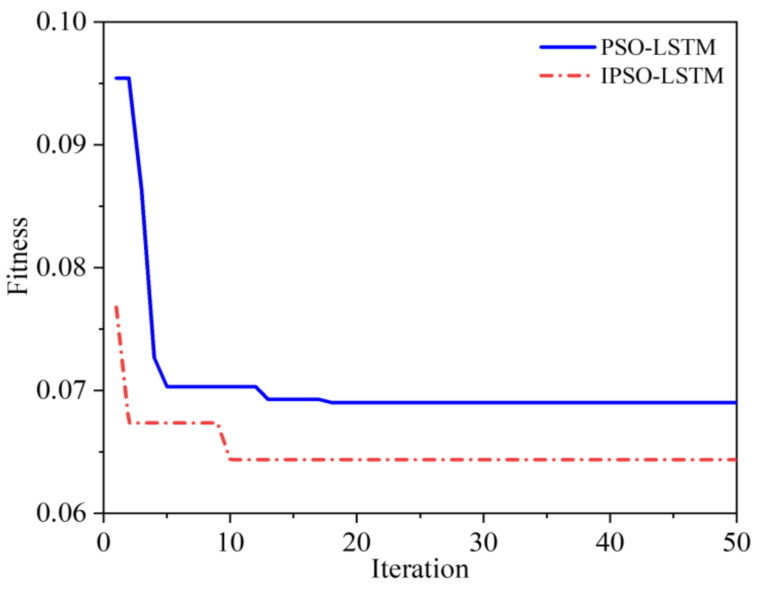
Optimization iteration results of roller bearing.

The results demonstrate that the predictive accuracy of the proposed method is greater than that of the other two methods. The RMSE results of LSTM, PSO-LSTM, and IPSO-LSTM are shown in Table 5. The iteration results of IPSO and PSO optimization are shown in Figure 16. It demonstrates that the IPSO algorithm converges earlier and is less likely to succumb to the local minimum problem, which is an advantage over the performance of the PSO.

Similar to case 1, extreme learning machines (ELM) and support vector regression (SVR) are compared with the proposed method.

**Figure 17:**
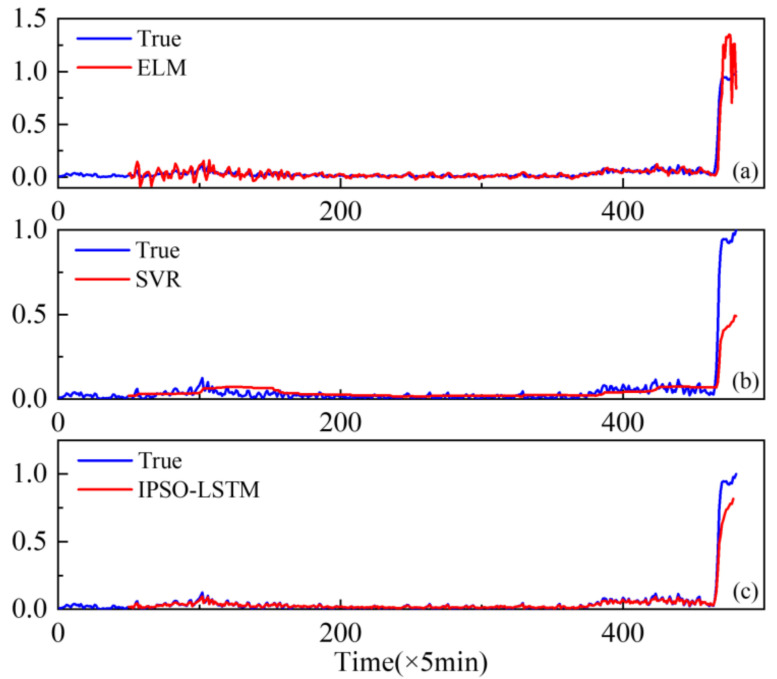
Performance degradation predictions of the roller bearing for the: (**a**) ELM; (**b**) SVR; (**c**) IPSO-LSTM.

The results of the comparison are shown in Figure 17 and Table 6. It can be seen that the proposed method is more effective than the other two methods in predicting the degradation trend of bearings. The RMSE values also reflect that the proposed IPSO-LSMT’s predictive accuracy is higher than the ELM and SVR methods.

## 5. Conclusions

This paper proposes a method based on an improved PSO optimized LSTM (IPSO-LSTM) to analyse bearing performance degradation. The proposed method can effectively resolve the problem of online parameter selection and the low predictive accuracy of the LSTM method. The KJADE method is used to fuse the bearing vibration signal to form an effective feature vector, and SS is calculated to acquire a performance degradation index. Then, the improved PSO algorithm is used to optimize the LSTM parameters to obtain an optimal performance degradation prediction model. In this study, the proposed method is compared with the LSTM, PSO-LSTM, ELM, and SVR through lab experiments. The experiments’ results have verified the effectiveness and superiority of the proposed method over others. This method has good prospective applications in predicting bearing performance degradation, and it can also be tailored and applied to other mechanical systems for online health and prognosis management.

## Figures and Tables

**Table 1:** Optimized Parameters.

Description	Notion
Number of nodes in the first LSTM layer	$h_{1}$
Number of nodes in the second LSTM layer	$h_{2}$
Batch size	S*m*

**Table 2:** Original features.

Time-domain	$T1=\frac{1}{N}\sum_{i=1}^{N}x_{i},$$\;\;\;T2=\sqrt{\frac{1}{N}\sum_{i=1}^{N}x_{i}^{2}}$$,\;\;\;\;T3={\left[\frac{1}{N}\sum_{i=1}^{N}\sqrt{\left|x_{i}\right|}\right]}^{2}$$,\;\;T4=\frac{1}{N}\sum_{i=1}^{N}\left|x_{i}\right|$,
	$T5=\frac{1}{N}\sum_{i=1}^{N}x_{i}^{3}$ $,\;\;T6=\frac{\sqrt{\frac{1}{N}\sum_{i=1}^{N}x_{i}^{2}}}{F4}$ $,\;\;\;\;T7=\frac{\max\left(x\right)}{\frac{1}{N}\sum_{i=1}^{N}\left|x_{i}\right|}$ $,\;\;\;\;T8=\frac{\frac{1}{N}\sum_{i=1}^{N}x_{i}^{4}}{{\left(\sqrt{\frac{1}{N}\sum_{j=1}^{N}x_{j}^{2}}\right)}^{4}}$
Frequency-domain	$F1=\frac{1}{N}\sum_{i=1}^{N}s_{i}$$,\;F2=\frac{1}{N}\sum_{j=1}^{N}(s_{j}-\frac{1}{N}\sum_{i=1}^{N}s_{i}{)}^{2}$$,\;F3=\frac{\sum_{i=1}^{N}f_{i}s_{i}}{\sum_{j=1}^{N}s_{j}}$$,\;F4=\frac{\frac{1}{N}\sum_{j=1}^{N}(s_{j}-\frac{1}{N}\sum_{i=1}^{N}s_{i}{)}^{3}}{{\left(\sqrt{F10}\right)}^{3}}$,
	$F5=\sqrt{\frac{\sum_{i=1}^{N}f_{i}^{2}s_{i}}{\sum_{j=1}^{N}s_{j}}}$ $,\;F6=\sqrt{\frac{1}{N}\sum_{i=1}^{N}s_{i}{\left(f_{i}-F12\right)}^{2}}$ $,\;F7=\sqrt{\frac{\sum_{i=1}^{N}f_{i}^{4}s_{i}}{\sum_{j=1}^{N}f_{j}^{2}s_{j}}}$ $,\;F8=\frac{\sum_{i=1}^{N}f_{i}^{2}s_{i}}{\sqrt{\sum_{j=1}^{N}s_{j}\sum_{k=1}^{N}f_{k}^{4}s_{k}}}$

**Table 3:** The RMSE of LSTM with different optimization methods.

RMSE	Outer	Roller	Inner
LSTM	0.042	0.039	0.042
PSO-LSTM	0.025	0.013	0.018
IPSO-LSTM	0.012	0.011	0.013

**Table 4:** The RMSE of different methods.

RMSE	Outer	Roller	Inner
ELM	0.055	0.031	0.029
SVR	0.031	0.027	0.029
IPSO-LSTM	0.012	0.011	0.013

**Table 5:** The RMSE of LSTM with different optimization methods.

	LSTM	PSO-LSTM	IPSO-LSTM
RMSE	0.065	0.054	0.048

**Table 6:** The RMSE of different methods.

	ELM	SVR	IPSO-LSTM
RMSE	0.073	0.101	0.048

## Data Availability

All data generated, or that appeared in this study, are available upon request by contact with the corresponding author. Furthermore, the models and codes used during the study cannot be shared at this time as the data also forms part of an ongoing study.
